# Novel Management for Severe Odontogenic Maxillary Sinusitis Based on Pathophysiology

**DOI:** 10.1155/2022/1614739

**Published:** 2022-07-27

**Authors:** Yoshio Ohyama, Masaru Ogawa, Satoshi Yokoo

**Affiliations:** ^1^Department of Oral and Maxillofacial Surgery, Shizuoka City Shizuoka Hospital, 10-93, Outemachi, Aoi-ku, Shizuoka-shi, Shizuoka 420-8690, Japan; ^2^Department of Oral and Maxillofacial Surgery, and Plastic Surgery, Gunma University Graduate School of Medicine, 3-39-15, Showamachi, Maebashi-Shi, Gunma 371-8511, Japan

## Abstract

Endoscopic sinus surgery is commonly performed to treat odontogenic maxillary sinusitis. However, recurrence and natural ostium reclosure often occur due to the inadequate patency of the excretory route. Furthermore, classical maxillary sinus radical surgery is still performed for odontogenic maxillary sinusitis even though it can cause postoperative maxillary sinus deformation, loss of function, and a postoperative maxillary cyst. A management that addresses these issues has not yet been identified. This study reported a conservative maxillary sinus management, wherein a nasoantral window is prepared and the thickened maxillary sinus mucosa is preserved, using the Caldwell–Luc approach. This study presents a case of severe odontogenic maxillary sinusitis that spread to the frontal sinus. This novel management facilitated complete recovery from severe odontogenic maxillary sinusitis in this case.

## 1. Introduction

Odontogenic maxillary sinusitis (OMS) management initially involved classical maxillary sinus radical surgery (MSRS) [1, 2]. However, this surgery was found to cause postoperative maxillary cysts (POMCs) several decades after treatment [3].

The maxillary sinus mucosa (MSM) is essential for the elimination of foreign substances and bacteria from the maxillary sinus. Removing the MSM during MSRS changes the anatomy of the maxillary sinus. Moreover, the maxillary sinus loses its function following the procedure [4]. Minimally invasive endoscopic sinus surgery (ESS) with natural ostium winding, which treats the causative tooth, has been commonly performed to treat OMS [2, 5]. However, ESS provides an inadequate excretory tract for the OMS, which is a more serious condition than general nasal sinusitis [4, 6]. In addition, the causative site of OMS is located distally, below the natural ostium. This study described a conservative maxillary sinus management (CMSM) without MSM removal for severe OMS spreading to the frontal sinus.

### 1.1. Case Report

**Figure 1:**
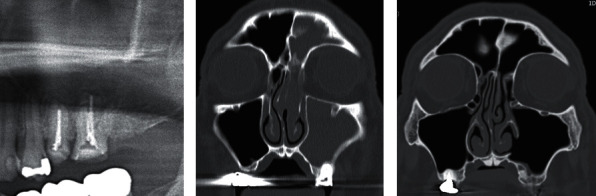
Radiographic findings. (a) Panoramic radiograph taken after the endodontic treatment of teeth #25 and #26 showing complete obturation of the roots except for the buccal distal root canal of #26. (b) Frontal plane of the preoperative computerized tomography (CT) scan revealed opacity extending from the left maxillary sinus to the left frontal sinus. (c) Frontal plane of the postoperative CT scan showed patency of the nasoantral window and the clearness of the left maxillary sinus and the left frontal sinus two years postoperatively.

A 54-year-old man was referred to our oral and maxillofacial surgery department by the otolaryngology department for OMS treatment. The patient came in with the chief complaint of pain radiating from his right cheek to his forehead. Physical examination revealed left cheek tenderness without swelling. Teeth #25 and #26 (FDI World Dental Federation ISO-3950) were root-canal-treated; however, the roots were underfilled. Panoramic radiograph suggested that the root apex was exposed to the maxillary sinus (Figure 1(a)). Computed tomography revealed opacification extending from the left maxillary sinus to the left frontal sinus (Figure 1(b)). The patient was clinically diagnosed with OMS caused by teeth #25 and #26. As the patient was keen to preserve his teeth, he was referred for endodontic treatment. His symptoms did not improve despite endodontic treatment and long-term antibiotic administration. Thus, CMSM was performed. Two years after the operation, recovery of OMS was observed. The anatomy and function of the maxillary sinus were completely restored. The nasoantral window was open, and the causative teeth were preserved (Figure 1(c)).

### 1.2. Surgical Procedure

**Figure 2:**
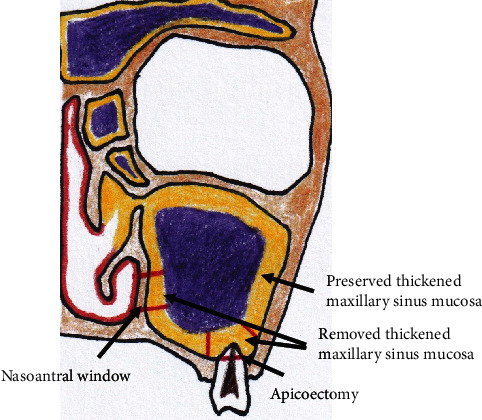
Schematic demonstration of CMSM. Apicoectomy was performed. A large nasoantral window in the inferior nasal meatus was prepared. The remainder of the thickened MSM was preserved.

Under general anesthesia, a 5- to 10-mm incision was made on the cervical side of the gingivobuccal fold of the left maxilla and the bone. The mucoperiosteal flap was raised, and the cortical bone around the canine fossa was removed to gain access to the left maxillary sinus. The root apices of teeth #25 and #26 protruded into the maxillary sinus, due to which the MSM corresponding to their root apices was removed through the left maxillary sinus followed by their apicoectomy (Figure 2). The root filling material of the buccal distal root canal of tooth #26 was not found (Figure 1(a)). Thus, retrograde filling was performed using 4-META/MMA-TBB resin. After irrigation with saline, the other thickened MSM was preserved (Figure 2). A large nasoantral window was prepared in the inferior nasal meatus. The vertical position of the nasoantral window was decided using the palatal wall as a guide. In addition, since the inferior nasal meatus was slightly raised on the inner wall of the maxillary sinus, the nasoantral window was created with a saw and hammer in this raised area atraumatically after removing the surrounding MSM. Finally, the nasoantral window was enlarged with the bone forceps (Figure 2). Gauze, with acromycin, was inserted into the maxillary sinus from the nasal cavity through the nasoantral window. The gauze was removed one week postoperatively. Clarithromycin was administered for one month postoperatively.

## 2. Discussion

MSM derived from pseudostratified ciliated epithelium is essential for maxillary sinus ventilation and clearance [7]. MSM removal, associated with MSRS, disrupts the normal function and morphology of the maxillary sinus [6, 8], resulting in POMC and sclerosis of the maxillary sinus wall. CMSM, developed by Yokoo et al. [4], preserves the MSM, facilitating the healing of the thickened pseudostratified ciliated epithelium and the recovery of maxillary sinus function. ESS has been commonly performed to treat OMS; however, ESS provides an inadequate excretory passage and ventilation hole, and recloses easily. In OMS, pus accumulates from the inferior nasal meatus due to gravity [6]. Therefore, a large nasoantral window in the inferior nasal meatus should be created to prevent nasolacrimal duct damage and to secure an excretory passage and ventilation hole [4]. Furthermore, apicoectomy and retrograde root canal filling of the causative teeth, which cannot be performed with ESS, can be achieved with CMSM. In this case, the function of the maxillary sinus and the causative teeth were successfully preserved via CMSM with apicoectomy and retrograde root canal filling. Further investigations involving a larger group of patients are necessary to establish this procedure as a treatment option.

## 3. Conclusions

This study proposed a novel treatment method, CMSM, which preserved the function and morphology of the maxillary sinus while preventing POMC in an OMS patient.
